# Malaria serology data from the Guiana shield: first insight in IgG antibody responses to *Plasmodium falciparum, Plasmodium vivax* and *Plasmodium malariae* antigens in Suriname

**DOI:** 10.1186/s12936-020-03434-y

**Published:** 2020-10-08

**Authors:** Mergiory Y. Labadie-Bracho, Farah T. van Genderen, Malti R. Adhin

**Affiliations:** 1 “Prof. Dr. Paul C. Flu” Institute for Biomedical Sciences, Kernkampweg 5, Paramaribo, Suriname; 2 Faculty of Medical Sciences, Department of Biochemistry, Anton de Kom Universiteit van Suriname, Kernkampweg 5, Paramaribo, Suriname

**Keywords:** Suriname, Guiana Shield, Seroprevalence, *Plasmodium*, Customized cut-off value, Antibody diversity, Multiplex bead assay

## Abstract

**Background:**

Suriname has accomplished a steep decline in malaria burden, even reaching elimination levels. *Plasmodium* serology data are not available for Suriname and even extremely scarce within the region, therefore malaria serology testing was introduced, country customized cut-off values were determined and a study was performed to explore the antibody status for *Plasmodium falciparum*, *Plasmodium vivax* and *Plasmodium malariae.*

**Methods:**

A cross-sectional survey was conducted between July 2017 and March 2018 in two areas of the interior with different malaria settings: Stoelmanseiland, representing Maroon villages and Benzdorp, a gold mining area, with mostly Brazilian miners. Dried blood spots (DBS) were collected (n = 197) and antibody presence against seven *Plasmodium* antigens was detected using a multiplex bead-based, IgG antibody assay. Demographic information was gathered through a questionnaire. Country customized cut-off values were generated from a Surinamese malaria-naïve reference population (n = 50).

**Results:**

Serological analysis for the reference population revealed cut-off values ranging from 14 MFI for LSA-1 to 177 MFI for PmMSP-1_19_. Seroprevalence against any of the three MSP-1_19_ antibodies was similar in both regions and surpassed 75%. Single seropositivity against PfMSP-1_19_ antibodies was higher in Stoelmanseiland (27.0%) than Benzdorp (9.3%), in line with the historical malaria burden of Stoelmanseiland, while the reverse was observed for PvMSP-1_19_ antibodies. Despite sporadic reports of *P. malariae* infections, PmMSP-1_19_ antibody presence was 39.6%. A more detailed examination of *P. falciparum* serology data displayed a higher seroprevalence in villagers (90.7%) than in Brazilians (64.6%) and a highly diverse antigenic response with 22 distinct antibody combinations.

**Conclusions:**

The results on the malaria antibody signature of Maroon villagers and Brazilian miners living in Suriname displayed a high *Plasmodium* seroprevalence, especially for *P. falciparum* in villagers, still reflecting the historical malaria burden. The seroprevalence data for both regions and the observed combinations of *P. falciparum* antibodies provided a valuable dataset from a historically important region to the international malaria serology knowledge. First insight in malaria serology data for Suriname indicated that the use of other target groups and assessment of age-dependent seroprevalence are required to successfully use malaria serology as tool in the national elimination strategy.

## Background

Malaria incidence and mortality have declined significantly in Suriname since 2006, due to stringent measures from the National Malaria Programme, such as the use of artemisinin combination therapy, widespread distribution of free long-lasting insecticide-impregnated mosquito nets, awareness campaigns and improved case management (i.e., active case detection (ACD), special programme for risk populations including tailored activities for miners scattered in the interior). A marked decrease was noted, particularly for *Plasmodium falciparum* infections, with a drop from more than 10,000 cases in 2001, to fewer than 800 cases since 2010. In April 2016, Suriname was identified by WHO (World Health Organization) as a country with the potential to reach elimination of local malaria transmission by 2020. However, the road towards malaria elimination is paved with challenges. Firstly, the looming threat of emerging artemisinin resistance. The Guiana Shield, comprised of the interior of Guyana, Suriname, French Guiana, and bordering areas of Brazil, Colombia and Venezuela, has been recognized by the PAHO (Pan American Health Organization) [1] as a region with an increased risk for the selection of resistant parasites because of the similarities with the Greater Mekong Sub-region, where resistance to artemisinin first emerged.

Indeed, several warning signals for emerging resistance were reported in studies conducted in Suriname. An in vivo efficacy study conducted in 2011 demonstrated that 31% of *P. falciparum*-infected patients, treated with Coartem^®^ were still positive on day 3 [2]. An in vivo efficacy study in 2014 with artesunate further revealed that at least 17.9% of samples exhibited a parasite half-life ≥ 5 h [3]. Molecular characterizations of drug-associated markers of isolates from Suriname did not reveal noteworthy alterations in potential molecular markers in the *pfATP6* [4], *pfmdr1* [5] and *K13* genes [6], but increased *pfmdr1* copy numbers in *P. falciparum* isolates [7] have been observed. The possible emerging of resistance to artemisinin is further fueled with reported self-treatment and poor adherence by miners in remote areas.

The second challenge in the process of malaria elimination is the country’s geographical location within the Guiana Shield, situated between two different, unfavourable epidemiological settings. In the east, French Guiana with a malaria prevalence of 22.3% in gold miners [8], and in the west, Guyana with increasing malaria and a control program struggling to provide services over a large area with miners in the mountainous forested areas near the border with Venezuela.

The majority of the cases of malaria, diagnosed and treated in Suriname in 2018, originated across borders, predominantly from French Guiana [9]. Spill-over from active foci from neighbouring countries will challenge all progress in Suriname, especially when the recent resistance history of the region is taken into account. French Guiana has reported in vitro resistance to artemether [10] and isolates from Guyana harboured the *C580Y* mutation in the *K13* gene [11]. Suriname is actively pushing forward a joint integrated approach for malaria interventions with the French, Guyanese and Brazilian governments.

Thirdly, malaria transmission occurs almost exclusively in hard-to-reach, mobile, migrant, artisanal gold mining populations in the remote areas of the interior and the illegal nature of the mining activities, on top of poor compliance with national treatment policies, further hampers the already logistically difficult and expensive operational processes.

Lastly, the lack of suitable monitoring procedures for malaria elimination, as most of the current field activities evolved from historical malaria control programmes, which are not yet optimized for low malaria numbers and a zero tolerance strategy.

These challenges underscore the need to implement innovative elimination strategies and appropriate tools and procedures for vigilant monitoring of every case in order to reach malaria elimination in the country. The common malaria detection tools, such as microscopy, rapid diagnostic tests and PCR detection, do not suffice for monitoring of elimination, especially within the mobile migrant mining population in the interior. Malaria serology alone or in conjunction with other malariometric indicators has proved to be a useful tool for malaria control and especially for malaria elimination programmes. It has been demonstrated that serological data can aid countries striving for malaria elimination through a better understanding of the transmission dynamics of the *Plasmodium* parasite especially in hypo-endemic malaria settings [12–14], where often long-lived antibody responses are easier to detect than asymptomatic or symptomatic malaria infections.

As malaria infection imposes a lasting antibody ‘footprint’, malaria serology data reflects cumulative malaria exposure and can serve various purposes. Serology data can be utilized to evaluate changes in exposure, to monitor long-term trends, besides geographic mapping and assessments of potential effects of control interventions. International studies in various geographical settings have been conducted to determine seroconversion rates (antibody acquisition rates of a population) and seroreversion rates (loss of antibody) from age-dependent seroprevalence data [12, 13]. However, *Plasmodium* serology data are not available for Suriname and are extremely scarce within the region.

This study aimed to gain first insight into the antibody status for the three circulating *Plasmodium* species (*P. falciparum, Plasmodium vivax* and *Plasmodium malariae*) in Suriname, with the introduction of a serological bead-based malaria multiplex assay, offering the possibility to simultaneously detect IgG antibodies against different *Plasmodium* species.

Country customized cut-off values were generated from a Surinamese malaria-naïve reference population and *Plasmodium* IgG distributions were analysed for persons from two distinctly different malaria environments: Stoelmanseiland, representing Maroon villages in Suriname, and Benzdorp with a mostly migrant gold mining population. Dried blood spots (DBS) and demographic information were collected from persons from both sites and serological assessment was performed using seven *Plasmodium* antibodies.

## Methods

### Study design, period and study site

In order to achieve a representative cut-off value for Suriname, a malaria-naïve reference population was generated, consisting of 50 adults living in Suriname (i.e., non-immune cohort) and cut-off values were calculated. Secondly, a cross-sectional investigation was conducted in two areas in the interior of Suriname with a different malaria environment, Stoelmanseiland and Benzdorp and their surroundings, to gain first insight in malaria seroprevalence in different regions in Suriname. Participant data and samples for serological testing were collected between July 2017 and March 2018. Thirdly, the historical significance of *P. falciparum* for the Guiana Shield prompted a more detailed investigation for the presence of *P. falciparum* antibodies in two sub-populations of interest: the Surinamese participants from Stoelmanseiland (n = 86) and the Brazilians from Benzdorp (n = 65).

### Study setting

Suriname is part of the Guiana Shield and is located along the northeastern coast of South America bordering French Guiana to the east, Guyana to the west and Brazil to the south. Suriname is the smallest sovereign country in South America with just over 575,000 inhabitants, most of whom live in the coastal area in and around the capital, Paramaribo [15]. Malaria transmission occurs in the interior, a tropical rainforest inhabited by Maroons and Amerindians living in communities near the main rivers. In recent years, mobile migrants, mainly nationals from Brazil, entered these regions, mostly via French Guiana for mining activities. Malaria-imported cases steadily increased and malaria transmission in Suriname is maintained mainly due to imported cases, which not only exceeded the national malaria burden, but accounted for more than 85% of the total malaria burden in 2018 [9]. Suriname recorded only 231 malaria cases in 2018, the majority caused by *P. vivax*.

*Anopheles darlingi* is the principal vector for malaria transmission. Historically, *P. falciparum* had been the predominant malaria species in Suriname until 2006, after which *P. vivax* became the main species. *Plasmodium malariae* has not been detected in Suriname since 2013.

The scarcity of serology data in the region necessitated the compilation of initial serology data, starting with the determination of country customized cut-off values and a subsequent investigation of variations in antibody status in persons from two regions with a different malaria environment.

### Stoelmanseiland and surroundings (Gakaba, Apoema and Jamaica)

Stoelmanseiland is a village located at the junction of Tapanahony River and Lawa River at the border of Suriname with French Guiana. Selection of this area was based on its historical malaria incidence, the highest in the period between 1999 and 2003, representing more than 10% of the overall national malaria burden. The inhabitants, mainly Maroons, live in small villages and camps along the river. Healthcare in this region is provided free for all by the Medical Mission and in 2016, the number of Stoelmanseiland inhabitants registered for healthcare was 1010. In the past 5 years however, an average of three cases (*P. falciparum* and *P. vivax*), including import cases was registered annually, by the National Malaria Programme.

### Benzdorp region

The Benzdorp region is a mining area in the interior with a population of approximately 2500–3000 persons in several settlements, some directly along Lawa River bordering French Guiana. This region was selected for the composition of the population, consisting of service providers and mobile gold miners, mainly Brazil nationals, entering the country primarily through French Guiana. The Malaria Programme of the Ministry of Health performs surveillance and provides all malaria services free of charge to these high-risk populations, supported by the Global Fund. Positive import cases (*P. falciparum* and *P. vivax*) are still recorded for this area, while diagnosis of indigenous malaria cases has been sporadic in the last 5years.

### Study population and data collection

Without existing data on antibody prevalence of the three *Plasmodium* species in Suriname, the minimum expected antibody prevalence was derived from reports from other countries in South America with similar malaria settings, such as Ecuador (48.8%) [16] and Pará State, Brazil (58.1%) [13]. The required sample size was determined based on an estimated average antibody prevalence of 53.5%, a 95% confidence interval and precision level of 10%, resulting in a minimum sample size of 89 and 93 persons for Stoelmanseiland and Benzdorp, respectively.

For both regions, all consenting adults (≥ 18 years), male or female, living or working in the respective areas were eligible for participation in the period between July 2017 and March 2018. Fever or microscopically confirmed malaria was applied as exclusion criteria. For the Benzdorp region, participants were recruited during ACD conducted by the National Malaria Programme. For Stoelmanseiland and surroundings, all villagers who could be reached within a 4-day enrolment period were requested to participate. In order to achieve a representative sample of actual villagers, Brazilians either entering via Brazil or French Guiana were excluded for this site.

Sampling and data collection were performed by trained field workers who visited each area alongside personnel from the National Malaria Programme or staff from the Medical Mission. Information about the study was provided in the native language of the participant through local translators. Participant information was gathered using an interviewer-administered, pre-tested questionnaire comprised of questions regarding basic demographic information and questions concerning history of malaria, mining and travel. Finger-prick dried blood spots (DBS) were collected onto Whatman^®^ 903 protein saver cards from all participants. Samples were individually packed in plastic ziplock bags containing desiccant and were transported from the interior at room temperature and subsequently stored at −20 °C until processing.

For the sub-set of participants from Benzdorp (n = 108), recruited during ACD, additional testing common during ACD, such as malaria rapid diagnostic tests (RDTs), thick and thin smears for malaria microscopy as well as real-time PCR for *Plasmodium* detection with photo-induced electron transfer (PET) primers [17] was performed.

### Immunoassay

Blood spot elution and immunoassays were performed according to protocols described elsewhere [14] with the following adjustments. From each DBS, three 3-mm circular punches were used for overnight elution at 4 °C. Antibody responses were measured using a magnetic Luminex^®^ bead-based multiplex immunoassay. IgG responses were measured for the merozoite surface protein-1_19_ antigens of *P. falciparum* (PfMSP-1_19_), *P. vivax* (PvMSP-1_19_) and *P. malariae* (PmMSP-1_19_). In addition, the responses for four other *P. falciparum* antigens: apical membrane antigen‐1 (AMA‐1), glutamate-rich protein (GLURP), circumsporozoite protein (CSP), and liver stage antigen-1 (LSA-1) were studied, next to tetanus-toxoid. The 19 kD fragment of the MSP-1_19_ was fused to glutathione S-transferase (GST), cloned and coupled to the beads by CDC, Atlanta (20 µg/ml beads for all three species). For AMA-1, produced at Walter Reed Army Institute of Research, 20 µg/ml beads; GLURP, CDC, 30 µg/ml beads; CSP, CDC, 30 µg/ml beads; LSA-1, CDC, 60 µg/ml beads and tetanus-toxoid, Massachusetts Biologic Laboratories, 12.5 µg/ml beads were used. The coupled beads were provided by V. Udhayakumar from CDC, Atlanta.

The selected *P. falciparum* antigens are all vaccine candidates and included two pre-erythrocytic and three blood-stage antigens. AMA‐1 and MSP-1_19_ are blood-stage antigens and both are considered to induce long-lived humoral immune responses after malaria infection. AMA-1 is highly immunogenic and has been suggested to be involved in red blood cell invasion by the merozoite, while MSP-1_19_ is regarded as moderately immunogenic. GLURP is a key merozoite surface antigen, which is expressed at each stage of parasite life in the human host. The pre-erythrocytic CSP and LSA-1 are considered to induce short-living antibodies.

A final dilution (1:100), from whole blood eluted for each sample from DBS, was incubated in flat-bottom plates with antigen-coupled BioPlex^®^ COOH beads (BioRad, Hercules, CA, USA) per analyte. Secondary antibodies were tagged with biotin (1:250 dilution anti-human IgG_1–3_ and 1:625 dilution anti-human IgG_4_, (Southern Biotech, Birmingham, AL, USA)) and incubated for 90 min at room temperature on a shaker. Subsequently, 50 μl of 1:200 dilution streptavidin, R–phycoerythrin conjugate (Invitrogen, Waltham, MA, USA) was added and incubated for 30 min at room temperature on a shaker. After various washing steps, beads were analysed on a MAGPIX^®^ system (Luminex, TX, USA) using the xPONENT 4.2 software for assay design and data acquisition. The reader was set to read a minimum of 50 beads/analyte of unique fluorescent signature per region.

In each experiment, positive and negative controls and all samples were run in duplicate to monitor intra-experimental variation. Pooled malaria-positive sera, obtained from V. Udhayakumar from CDC Atlanta, served as positive control to assess day-to-day variability, while the negative control consisted of an eluate from blank filter paper. Blanks (PBS) were also included to determine the background signal within each run. The median fluorescence intensity (MFI) as output for each sample was calculated as the mean value of the duplicate wells after subtraction of MFI values originating from the blank background beads (MFI-bg). The net MFI was calculated by subtracting the respective cut-off value from the MFI-bg. Retesting was performed for samples with poor elution, or for replicates with a CV higher than 20% for their MFI-bg values.

### Determination of country customized cut-off value

The majority of malaria serology studies utilize cut-off values either obtained from ELISA or earlier reported values for multiplex assays, derived from non-immune reference populations, consisting of US citizens with no travel history to malaria-endemic countries [14]. More recently, study-specific cut-offs are generally determined under the particular study conditions, but usually still using a panel of samples from US residents, without history of travel outside the USA [18].

However, conditions in Suriname are quite different, with a highly multi-ethnic population, distinctive population genetics, heterogeneous malaria history, varying environments (Maroon villages, Amerindian settlements and mining areas) and a dissimilar distribution of *Plasmodium* species.

Therefore, a malaria-naïve reference population was generated, consisting of 50 adults living in the coastal area of Suriname for at least 10 years, with no fever and no reported history of malaria infection (i.e., non-immune cohort).

The background signal intensity for the DBS from the non-immune reference cohort, eluted and processed within the study setting was utilized to determine the specific background signal (customized cut-off value) for each of the seven *Plasmodium* antigens. Determination of the ‘true negative’ serodistribution for Suriname was calculated for each antigen as the mean signal from the non-immune reference population for the respective antigen without outliers + 3SD, according to calculations described elsewhere [14].

The acquired cut-off values were applied for the corresponding study results, thus generating the true seropositivity value for each experiment. In addition to the seven *Plasmodium* antibodies, tetanus-toxoid was included to serve as possible internal positive control for the non-immune population, as substantial responses to this antigen were expected, based on the high national immunization coverage.

### Statistical analysis

The Chi square test was used to study associations between malaria antibodies and variables, such as history of malaria and travel, and for seroprevalence comparison between groups. Logistic regression was used to correlate seropositivity against PfMSP-1_19_ and PmMSP-1_19_ antibodies with age. The data were analysed using Statistical Packages for Social Sciences (SPSS version 22.0). Statistical significance was set at p < 0.05.

## Results

### Country customized cut-off values

**Fig. 1:**
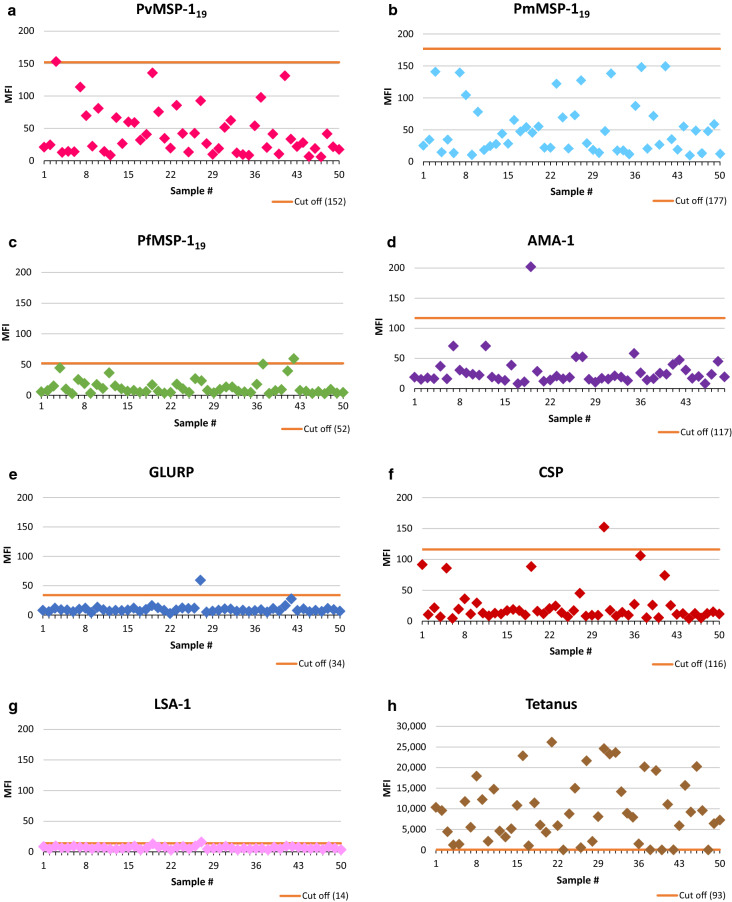
Distribution of MFI values within the malaria-naïve cohort. Scatter plots showing the distribution of MFI values for all tested malaria antibodies from the non-immune cohort (panel **a-g**). MFI values represent the mean value of the sample duplicates after subtraction of MFI values originating from the blank background beads (MFI-bg). Mean blank background MFI values and MFI range in brackets: PfMSP-1_19_: 16 [16, 17]; PvMSP-1_19_: 20 [19–21]; PmMSP-1_19_: 18 [17–19]; AMA-1: 19 [16–22]; GLURP: 13 [12–15]; CSP: 33 [32, 33] and LSA-1: 14 [14, 15]. The horizontal line represents the calculated cut-off of the respective antibody. One outlier for AMA-1 (MFI: 974) was excluded from the calculations. A scatter plot for tetanus-toxoid antibodies in this malaria reference population is also shown in panel **h**

Serological analysis for the non-immune cohort (n = 50) revealed no anti-malarial antibodies as expected, and the generated values validated the selection criteria used for the non-immune Surinamese cohort. Calculation of the true negative serodistribution for each malaria antigen resulted in the cut-off values depicted in Fig. 1 panel A-G.

The *Plasmodium* antibody cut-off values ranged from 14 for LSA-1 to 177 for PmMSP-1_19_. The cut-off value determined for *P. falciparum* MSP-1_19_ antibodies was lower than those for *P. malariae* and *P. vivax.* The presence of tetanus-toxoid antibodies in the malaria non-immune participants from Suriname was 90.0%, coinciding with the WHO estimated immunization coverage for Suriname in 2018 for DPT1/DPT3 (95%) [19]. Tetanus-specific antibody levels were high, averaging 10,533 MFI (minimum: 500 MFI and maximum 26,078 MFI) in the tetanus positive population (Fig. 1 panel H).

### General malaria serology aspects

Serological analysis for the seven utilized *Plasmodium* antigens (LSA-1, GLURP, CSP, AMA-1, PfMSP-1_19_, PvMSP-1_19_, PmMSP-1_19_) was successfully conducted for all collected blood samples (n = 197). The detected antibody intensities were highly variable for the seven malaria analytes and positive MFI values even exceeded 30,000, both for PfMSP-1_19_ and AMA-1. The presence of *Plasmodium* antibodies was positively linked to the documented number of infections, in line with the common belief that cumulative exposure will elicit a long-lived response. Data on malaria history revealed that the majority of participants (75.6%) reported one or more past malaria episodes, of which only 21.5% could recollect the responsible *Plasmodium* species. Furthermore, 70.5% of the participants with a malaria history reported multiple infections, some surpassing more than 20 infections during their lifetime. The results revealed a positive association for all seven *Plasmodium* antigens between self-reported malaria history and actual presence of *Plasmodium* antibodies (p < 0.05). However, the timeline of previous malaria episodes of the participants and the correlation with the current presence of antibodies could not be examined, since information was gathered on the number of past episodes, but details were only collected for the most recent infection, anticipating recall bias.

The correlation of the seropositivity with age was studied only in the Surinamese participants from Stoelmanseiland for PfMSP-1_19_ antibodies. The low presence of *P. vivax* antibodies in the mainly Maroon population in Stoelmanseiland prohibited this analysis for *P. vivax* antibodies. Age was positively associated with seropositivity against PfMSP-1_19_ antibodies for every 10-year increase, in age increments between 20 and 69 years. The odds ratio per 10-year increase was 1.7 (CI 95% = 1.1–2.6, p < 0.05). The lack of all age groups within this study population prohibited the determination of seroconversion rates.

*Plasmodium vivax* parasites seem to require the Duffy Antigen Receptor for Chemokines on the erythrocytes for their attachment and *P. vivax* infection should therefore be uncommon in persons with the Duffy-negative (FyFy) blood group, which is widespread in populations with African ancestors. To investigate this concept, the total population with known ancestry was grouped as participants from African descent (n = 124) and participants without African ancestry (n = 63). Indeed, general occurrence of *P. vivax* antibodies was significantly lower in persons from African descent (p < 0.001).

### *Plasmodium* MSP-1_19_ presence in Stoelmanseiland *versus* Benzdorp

**Fig. 2:**
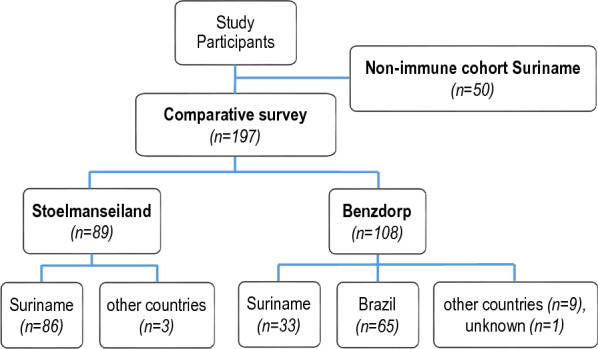
Overview of the study participants. The comparative survey was performed between two populations with different malaria history: villagers (Stoelmanseiland) and migrant miners (Benzdorp). The composition of each population based on country of origin is represented

An overview of the 197 participants enrolled for the comparative investigation is presented in Fig. 2. The survey population for Stoelmanseiland (n = 89) was predominantly comprised of participants born in Suriname (n = 86), while the Benzdorp population (n = 108) consisted mainly of migrants, with the majority from Brazil (n = 65), followed by Dominican Republic nationals (n = 5), French Guiana (n = 2), Philippines (n = 1), and Venezuela (n = 1) and data were missing for one participant (n = 1). Benzdorp participants were recruited during ACD and malaria diagnostics, including quantitative molecular *Plasmodium* detection revealed no *Plasmodium* presence in any of these participants. The mean age of the study population was similar for both areas (Table 1).

**Table 1:** Characteristics of participants from the Stoelmanseiland area and the Benzdorp region

	Stoelmanseiland area	Benzdorp region
	*(n *= *89)*	*(n *= *108)*
*Age in years*		
Mean [95% CI]	39.7 [37.3–42.2]	41.4 [39.5–43.3]
SD	11.6	10.0
n	88	105
*Gender*^a^		
Male	16.9%	75.9%
Female	80.9%	24.1%
*History of malaria*^b^		
Yes	82.0%	70.4%
No	18.0%	29.6%

^a^Missing data in Stoelmanseiland = 2

^b^Lifetime history

The ratio of males to females (M/F) for the Benzdorp population was higher than the general Surinamese population (M/F: 3.15 *vs* 1.0) as could be anticipated with mostly male participants in gold mining activities. On the other hand, the gender distribution for the Stoelmanseiland area (M/F: 0.21) was skewed towards women, which could be for a number of reasons including differences in earlier documented health-seeking behaviour, the perceived reluctance of local men to participate in investigations, especially when blood collection is required and the sampling during day time when the majority of men were engaged in activities (e.g., hunting, fishing, mining) outside the village.

The representation of ethnic groups was not in line with the country’s ethnic distribution, as could be expected with enrolment in the interior. The over-representation of Maroons in the Stoelmanseiland area was 92.1 *versus* 22.0% in the general Surinamese population. For the Benzdorp region, Brazilians accounted for 60.2% of the enrollees.

**Fig. 3:**
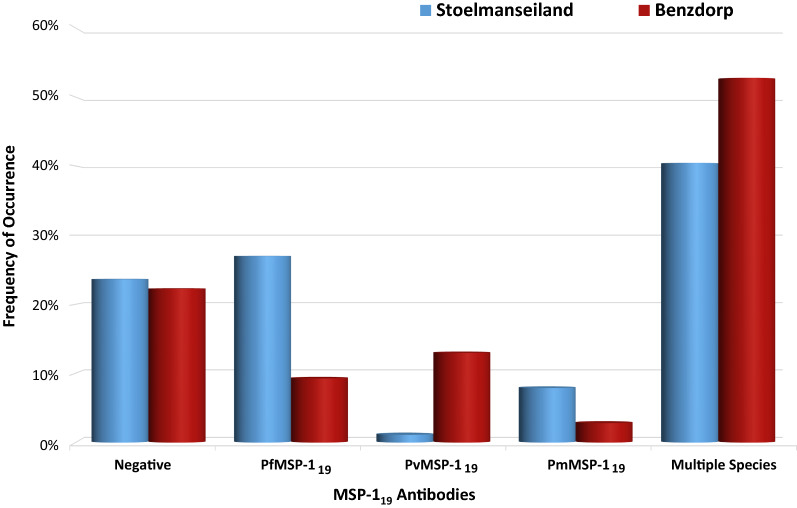
*Plasmodium* MSP-1_19_ antibody prevalence in participants from Stoelmanseiland and Benzdorp. The three clustered columns in the middle represent single MSP-1_19_ antibody prevalence, while the last clustered columns depict presence of more than one MSP-1_19_ antibody for both populations

**Fig. 4:**
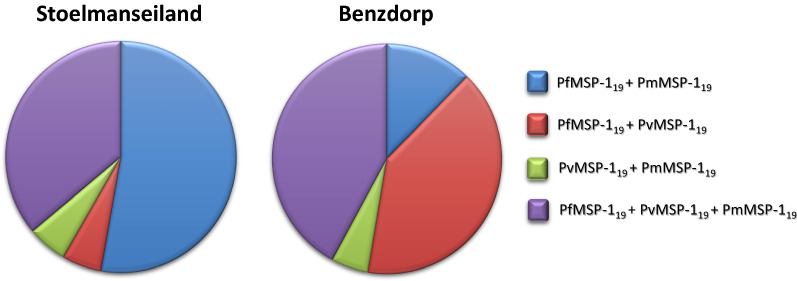
Distribution of MSP-1_19_ antibodies against multiple *Plasmodium* species in participants from Stoelmanseiland and Benzdorp. Any combination of multiple species MSP-1_19_ antibodies is depicted for both regions, with a different colour for each combination

In the comparison of seroprevalence between Stoelmanseiland and Benzdorp, with their diverging malaria environment, only the three MSP-1_19_ antibodies (*P. vivax, P. falciparum* and *P. malariae*) were evaluated, to prevent skewing towards *P. falciparum.* The distribution of MFI values for each of the antibodies used in this comparative survey is shown in the Additional file 1: Figure S1. Overall seropositivity against any of the three MSP-1_19_ antibodies was similar in both regions and surpassed 75%. The prevalence of antibodies against more than one *Plasmodium* species was higher than single-species antibody prevalence in both regions. The *Plasmodium* MSP-1_19_ antibody prevalence in participants from Stoelmanseiland and Benzdorp is illustrated in Fig. 3, while the distribution of multiple species seropositivity is depicted in Fig. 4.

Single-species seropositivity against PfMSP-1_19_ antibodies displayed a significantly higher occurrence in Stoelmanseiland (27.0%) *versus* Benzdorp region (9.3%) (p < 0.05), while the reverse was observed for PvMSP-1_19_ antibodies (p < 0.001) (Fig. 3). The PvMSP-1_19_ prevalence in Benzdorp was even higher, if Brazilian miners only from this region were considered (41.7%). The single-species *P. malariae* seroprevalence did not exhibit a significant difference between Stoelmanseiland and Benzorp participants (p = 0.182). The overall multiple-species antibody presence did not exhibit a significant variation within the two regions (p = 0.374) (Fig. 3), although significant differences existed in the distribution of the combinations (Fig. 4).

In Stoelmanseiland, MSP-1_19_ *P. falciparum/P. malariae* was the leading mix (52.8%), while the *P. falciparum/P. vivax* combination was more common in Benzdorp (40.4%).

Interestingly, 20.7% of the Surinamese Maroon population in Stoelmanseiland harboured *P. vivax* antibodies in combination with other malaria species antibodies, even though carriage of single species antibodies *to P. vivax* was only observed in one participant from Stoelmanseiland (Fig. 3).

The majority of PmMSP-1_19_ (87.2%) was observed in conjunction with antibodies against other malaria species (Fig. 4) and the presence of PmMSP-1_19_ antibodies in 39.6% of all study participants was a remarkable finding, in light of the infrequent *P. malariae* infections registered in South America.

Possible impact of cross reactions with other *Plasmodium* antibodies did not seem likely, since high MFI levels of *P. falciparum* and *P. vivax* antibodies were observed without any *P. malariae* antibodies, while four participants exhibited the sole presence of *P. malariae* antibodies.

Antibodies against all three *Plasmodium* species (*P. falciparum, P. vivax* and *P. malariae*) were more often detected in Benzdorp (22.2%) than in Stoelmanseiland (14.6%), although not reaching significance (p = 0.292).

Travel to a neighbouring country within the past 5 years was reported by 55.8% of all participants. Analysis of the seropositivity against MSP-1_19_ antibodies revealed that travel was not associated with the presence of PvMSP-1_19_, PfMSP-1_19_ and PmMSP-1_19_ antibodies in Stoelmanseiland. In Benzdorp, travel was associated only with the presence of PfMSP-1_19_ antibodies and more specifically for the sub-group (n = 33) of mobile Surinamese miners in this region (p < 0.05).

### *Plasmodium falciparum* seroprevalence

The historical significance of *P. falciparum* within Suriname and the Guiana Shield, alongside the expectation that elimination of *P. falciparum* will precede elimination of the relapse-prone *P. vivax* species, prompted a more detailed examination of *P. falciparum* serology data with five antigens for two sub-populations of interest, namely Surinamese villagers from Stoelmanseiland and Brazilian miners from the Benzdorp region (Table 2).

**Table 2:** Overall seropositivity for IgG *Plasmodium falciparum* antibodies in Surinamese and Brazilian participants in Suriname

	Surinamese participants^a^ (%)	Brazilian participants^b^(%)	*P* value ^c^
*P. falciparum* seropositivity ^*d*^	90.7	64.6	*<0.001*
AMA-1	72.1	52.3	*<0.05*
PfMSP-1_19_	66.3	60.0	0.427
GLURP	62.8	41.5	*<0.05*
CSP	22.1	24.6	0.716
LSA	38.4	16.9	*<0.05*

^**a**^Surinamese participants from the Stoelmanseiland area (n = 86)

^**b**^Brazilian participants from the Benzdorp region (n = 65)

^**c**^p-values for differences in seropositivity between Surinamese and Brazilian participants

^**d**^Detection of at least one of the five investigated *P. falciparum* IgG antibodies

**Fig. 5:**
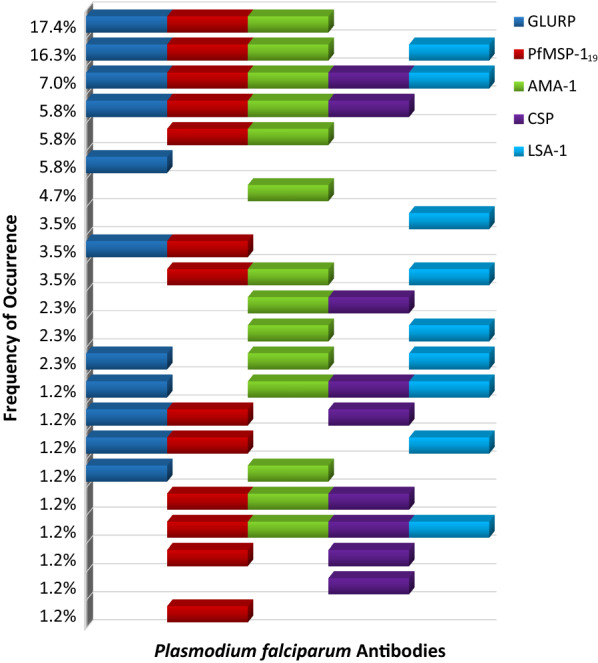
Frequency of occurrence of different *Plasmodium falciparum* antibody combinations in Surinamese participants from Stoelmanseiland. Each horizontal line represents a combination and the depicted coloured blocks in the line correspond with the comprising *P. falciparum* antibodies in the combination, Samples positive for *P. falciparum* antibodies (90.7%)

The *P. falciparum* seropositivity (detection of at least one of the five IgG antibodies) was statistically higher for the Surinamese participants compared to the Brazilians, as was the presence of the frequently investigated PfMSP-1_19_ and AMA-1, either alone or in combination (80.2 *vs* 63.1%, p < 0.05). The long-lasting antibodies were leading the overall prevalence in both sub-groups, while the presence of LSA-1 and CSP antibodies was significantly lower (p < 0.001). To gain some insight into the combined occurrence of specific long- and short-lasting *P. falciparum* antibodies in Suriname, all possible combinations of antibodies in the Surinamese participants from Stoelmanseiland were analysed. Twenty-two distinctive patterns were detected and the various combinations with their frequency of occurrence are depicted in Fig. 5.

In accordance with the high overall seroprevalence of the long-lasting antibodies, the two leading combinations consisted of the three long-lasting antibodies with and without LSA-1. As expected, the presence of long-lasting antibodies was detected in 86.0% of the participants, but despite a high prevalence for all three long-lasting antibodies, only 54.1, 25.7 and 20.3% of these positive cases carried all three, two or just one of these predominant antibodies, respectively. Furthermore, no correlation could be established between the two short-lasting antibodies CSP and LSA-1.

## Discussion

The current study provided the first malaria seroprevalence data for the three circulating *Plasmodium* species (*P. vivax, P. falciparum* and *P. malariae*) in Suriname, a country in the Guiana Shield with low malaria transmission in the last decade. Country customized cut-off values were determined for antibodies against seven common *Plasmodium* antigens, applicable for the utilized multiplex assay and to counteract population-specific differences in immune responses. The use of a reference population of Surinamese with no malaria history, instead of the general reference population consisting of US citizens with no travel history to malaria-endemic countries, enabled the generation of a true representation of the actual ‘negative’ serodistribution in this setting. The generated cut-off values in this study were lower than cut-off values for the commonly used reference population consisting of US citizens [20], probably due to the low background of the immunoassay and differences related to the coupling of the beads. However, even lower cut-off values have been demonstrated in a study setting in Haiti among 247 US citizens [14]. The observed lower cut-off value for MSP-1_19_ for *P. falciparum* compared to MSP-1_19_ for the other two *Plasmodium* species was in line with recent results [20].

Prior to 2006, Suriname had a very high malaria burden and was even considered as the country in the Americas with the highest concentration of falciparum malaria [21], which was reflected in the higher *P. falciparum* seroprevalence in villagers from Stoelmanseiland compared to the mobile migrants from the Benzdorp region. The expectations that both malaria history and higher age of an individual would be associated with presence of antibodies could be confirmed. The observed positive association between age and seropositivity against PfMSP-1_19_ antibodies for every 10-year increase in the Surinamese participants substantiated the receding local malaria transmission trend in Stoelmanseiland. Nevertheless, *P. falciparum* IgG positivity was still very high in Stoelmanseiland, despite scarce reports during the last decade. This outcome pointed to a slow decay of these antibodies and could be used to extrapolate the longevity of the *P. falciparum* antibody responses in this population in the absence of re-infection. However, it should be noted that antibody half-lives increase with age and that age is related to the presence of long-lived IgG antibody secreting cells that can maintain a sustained *P. falciparum* antibody response in the absence of re-infection [22].

The finding that a travel history to a malaria-endemic country was a risk-factor for *P. falciparum* only in mobile Surinamese miners from Benzdorp, in concert with the increasing trend of imported malaria cases in Suriname, highlighted the importance of keeping detailed records of patient travel history.

The results on seroprevalence of the three circulating *Plasmodium* species are further discussed in more detail per species. Comparative serological analysis is quite complex and malaria serology prevalence is influenced by a variety of factors, including the dynamic mechanism of antibody formation and decay for each antibody type, the target population, the geographic location, and the historical malaria burden of the area. Furthermore, available data of possible serological biomarkers of malaria transmission intensity have to be retrieved from studies conducted in quite different endemic settings, each employing only a few of the assortment of *Plasmodium* antibodies. Caution is therefore warranted for associations and comparisons, as confounding conclusions can be reached, if only a single mechanism aspect is considered.

The overall seroprevalence of the highly immunogenic PvMSP-1_19_ in Brazilian miners in the Benzdorp region (64.6%) was in line with reports from the Brazilian Amazon, where nearly 70% of the studied population carried PvMSP-1_19_ antibodies [23]. In accordance with the observation in this study that travel did not correlate with levels of PvMSP-1_19_ antibodies, relatively uniform seropositivity against PvMSP-1_19_ has been reported in the Brazilian Amazon, with or without travel between areas with markedly different malaria transmission levels [24].

The significantly lower occurrence of *P. vivax* antibodies detected in persons from African descent provided support for the general belief that *P. vivax* parasites use the Duffy Antigen Receptor for Chemokines on the erythrocytes for their attachment. The results that *P. vivax* antibodies were still detected in more than 20% of the Maroon population from Suriname seem to concur with the observation from the African West Coast that the Duffy-negative antigen no longer seems to be an insurmountable barrier for *P. vivax* merozoites [25]. On the other hand, it should be noted that the Maroons from the Stoelmanseiland area only harboured *P. vivax* antibodies in conjunction with other *Plasmodium* antibodies with just one exception. This finding could also suggest that Maroons living in co-endemic regions may have an increased risk of a *P. vivax* malaria infection, following another malaria episode, which augmented an earlier hypothesis that *P. falciparum* infections are a risk factor for *P. vivax* relapses in co-endemic regions [26].

The remarkably high presence of antibodies against *P. malariae* (39.6%) was in contrast with the sporadic *P. malariae* infections registered in South America. In the last decade, *P. malariae* infections rarely surpassed 1–2% in South America [27]. International reports record 0.1% for Brazil, Colombia, Costa Rica, French Guiana, Guyana, Peru, and Venezuela. However, the current serology data are more in line with studies in Colombia and Brazil, where nested PCR detection of symptomatic patients revealed a prevalence of *P. malariae* infections of 43.8 [28] and 11.9%, respectively [29]. The study results suggested that *P. malariae* infections may have long been under-detected in Suriname, due to difficult microscopic differentiation, although it should be noted that *P. malariae* infections can be long-lasting, even up to 40 years without re-infection [30]. The lack of a correlation between age and seroprevalence for PmMSP-1_19_ provided additional support for a reflection of archaic history. The observation that the majority of PmMSP-1_19_ antibodies (94.9%) appeared in conjunction with other malaria antibodies, corroborated results from Zimbabwe, where the frequency of IgG responses to both PmMSP-1_19_ and PfMSP-1_19_ ranged between 78 and 87% [31]. Possible impact of cross reactions with other *Plasmodium* antibodies was regarded unlikely as it has been demonstrated that IgG antibody responses to the utilized malaria MSP-1_19_ antigens appear to be species-specific, even in high transmission settings with multiple circulating species [18], besides the finding of four study participants harbouring single-species *P. malariae* antibodies.

Internationally, several findings on *P. falciparum* malaria serology prevalence have been reported, but originating from different endemic settings and achieved with varying types and numbers of antibodies tested, evidenced by the heterogeneity of published data. In this first malaria serology study in Suriname, a highly diverse antigenic response against *P. falciparum* was revealed, with an assortment of distinct combinations of long-lasting and short-lasting antibodies. The observed overall *P. falciparum* antibodies prevalence (IgG positivity for any of five tested *P. falciparum* antibodies) was significantly higher in the Stoelmanseiland area (90.7%) than in the Brazilian participants (64.6%, p < 0.001) and also substantially higher than the 24.7% from the neighbouring state of Pará in northern Brazil, although their IgG positivity results were obtained by testing only PfMSP-1_19_ and AMA-1 [13]. The disparity remained, if only the seropositivity against these two antigens (80.2%) was considered. The historical malaria burden of Suriname, with the highest recorded Annual Parasite Index (212.2 per 1000 population at risk) in the Americas in 2000, was despite the current low transmission settings, still mirrored in the extensive *P. falciparum* seropositivity in the population from Stoelmanseiland. The assessment of five different *P. falciparum* antibodies allowed for a more scrutinized examination of the combined occurrence of the long- and short-lasting antibodies in the Surinamese participants from Stoelmanseiland. The observed predominance of AMA-1, PfMSP-1_19_ and GLURP in the patterns reflected the high *P. falciparum* burden of the Surinamese population and the ability of the corresponding antigens to elicit long-lived humoral immune responses after malaria infection.

An uneven distribution of antibodies against the PfMSP-1_19_ and AMA-1 antigens was shown, with just one of these two long-lasting antibodies present in 22.1% of the cases. Other studies also observed a skewed antibodies distribution [12, 32]. In contrast to many studies, focusing only on AMA-1 and PfMSP-1_19_, the use of multiplex serology allowed simultaneous examination of GLURP with the two other long-lasting antibodies. The seroprevalence of GLURP in the Surinamese sub-group (62.8%) was in line with a Brazilian study on GLURP antibody response revealing a high prevalence (79.0%) [33], and did not substantiate the premise that GLURP antibodies reflect a recent (6 months) malaria exposure, as proposed in a study in the Greater Mekong Sub-region [34].

The mere presence in the study population of antibodies against the non-erythrocytic short-lived CSP with an estimated half-life ranging from 3 months [35] to about a year was somewhat troubling. Besides the low transmission numbers in Suriname, none of the participants had reported any symptoms and all available real-time PCR results were *P. falciparum* negative. Nonetheless, others also report the presence of CSP antibodies in persons long after malaria infection and even postulated that CSP antibodies, despite their short-lived nature, do not necessarily correlate with recent exposure [36].

Despite the insight gained in the various combinations of the long-, and short-lasting antibodies exhibited in this study group, the intricate aspects regarding lasting presence of specific antibodies challenged straightforward conclusions.

The malaria seroprevalence data in this study added to the international knowledge of different *Plasmodium* antibodies and their combinations and provided first insight into the antibody status of two different populations in Suriname (Maroon villagers and Brazilian miners). The high antibody response for the three long-lasting *P. falciparum* antibodies indicated an earlier antibody saturation, thus suggesting a limited value for these antibodies to detect significant recent transmission shifts in similar study settings with a heterogeneous historic malaria burden. However, the newly introduced serological assay in the country paved the way for future studies, possibly even using MSP-1_19_, but with different target groups, as it has been demonstrated that utilization of MSP-1_19_ in age-stratified surveys in northeastern Tanzania allowed differentiation of hyper- to hypo-endemic villages [37]. Moreover, assessment of age-dependent seroprevalence in various geographical areas may provide the malaria programme with a valuable asset, as seroconversion rate has been reported as a reliable tool for assessing the malaria endemicity of *P. vivax* and *P. falciparum* [13].

The major strength of this cross-sectional study is the presentation of first data on both short- and long-lasting *Plasmodium*-specific antibodies from persons living in the interior of Suriname. Moreover, serological data are reported for persons from two countries, Surinamese (mainly Maroon villagers) and Brazilian miners. Other strengths of the study are the determination of representative cut-off values for *Plasmodium* antibodies for Suriname and providing some insight in profile patterns of *P. falciparum* antibodies.

The study has limitations. First, data on malaria history, number of infections and *Plasmodium* species were obtained from self-reports of participants, probably resulting in recall bias. However, there are no indications that any recall bias would be different amongst participants from both areas. Second, the link between the burden of consecutive infections and malaria antibodies could not be addressed properly, since details of each previous malaria infection were unavailable. Third, the enrolment of only adults limited the extent of the gathered information and did not enable estimation of seroconversion rates. Lastly, this study was performed in just two study areas in the interior of Suriname. Hence, care should be taken to draw conclusions about malaria serology regarding children or persons residing in other geographic areas with historically different temporal malaria transmission. Follow-up studies in other areas with varying malaria histories and the inclusion of children are required to estimate seroconversion rates, and studies among other ethnic groups, such as the Amerindians, will provide further insight into the complex malaria serology in Suriname. Nevertheless, the current study results contributed to the international serology knowledge on malaria antibodies and introduced preliminary requirements for possible future use of this tool in Suriname for stratification of absence of transmission per region, which is particularly important for other countries also striving for malaria elimination.

## Conclusions

Prevalence of indigenous malaria has declined dramatically over the past years in Suriname, transforming the country from the area with the highest concentration of falciparum malaria in the Americas, to a country aiming for elimination. A multiplex bead-based assay was introduced and country customized cut-off values were determined, enabling future studies to utilize this panel of samples from the country specific malaria-naïve reference population. A cross-sectional seroprevalence study was conducted in two regions with a differing malaria environment. Higher *P. falciparum* MSP-1_19_ seropositivity was recorded in the Stoelmanseiland area, in line with the historical malaria burden of this region, while the reverse was demonstrated for *P. vivax* antibodies, presumably because of the high proportion of Maroons in Stoelmanseiland. The finding that the Maroons from Stoelmanseiland harboured *P. vivax* antibodies predominantly in conjunction with other *Plasmodium* antibodies suggested that Maroons living in co-endemic regions may have an increased risk of a vivax malaria infection, following another malaria episode.

The high presence of antibodies against *P. malariae* added to the data of the region that *P. malariae* infections may have long been under-detected in South America.

This study not only presented the first results on malaria and tetanus-toxoid antibody presence in Suriname, but the highly diverse profile patterns of *P. falciparum* antibodies with a multitude of combinations of long-lasting and short-lasting antibodies provided a valuable dataset from a historically important region to the international *P. falciparum* malaria serology knowledge. The high *P. falciparum* antibody response measured in the low transmission setting implied that a serological approach with any of the five studied antibodies may not detect significant recent deviations from the historic pattern of transmission in similar study settings. Additional studies with other target groups and assessment of age-dependent seroprevalence in other geographical areas of the country are required, prior to introduction of malaria serology as integral part of a multi-pronged elimination strategy for Suriname.

## Supplementary information

**Additional file 1: Fig S1.** Distribution of MFI values for MSP-1_19_ antibodies in Benzdorp and Stoelmanseiland.

## Data Availability

Additional file 1: Figure S1 Scatterplots illustrating the distribution of MFI values for MSP-1_19_ antibodies in the comparative survey accompanies this paper. The datasets generated and/or analysed during the current study are available from the corresponding author on reasonable request.

## Abbreviations

ACD: Active case detection
AMA-1: Apical membrane antigen‐1
CDC: Centers for Disease Control and Prevention
CSP: Circumsporozoite protein
DBS: Dried blood spot
GLURP: Glutamate-rich protein
LSA-1: Liver stage antigen-1
MFI: Median fluorescence intensity
MFI – bg: Median fluorescence intensity minus background
PBS: Phosphate-buffered Saline
PfMSP-1_19_: Merozoite surface protein-1_19_ antigens of *P. falciparum*
PmMSP-1_19_: Merozoite surface protein-1_19_ antigens of *P. malariae*
PvMSP-1_19_: Merozoite surface protein-1_19_ antigens of *P. vivax*
WHO: World Health Organization
PAHO: Pan American Health Organization
pfATP6: *P. falciparum Ca*^*2*^+-*ATPase* Coding gene
PfMDR1: *P. falciparum* Multidrug resistance transporter 1
