# Supplementary material for: Malaria serology data from the Guiana shield: first insight in IgG antibody responses to Plasmodium falciparum, Plasmodium vivax and Plasmodium malariae antigens in Suriname
Source: Malar J. 2020 Oct 8;19:360. doi: 10.1186/s12936-020-03434-y (PMC7545893; doi:10.1186/s12936-020-03434-y)
Supplement: Supplementary file 1 — Additional file 1: Fig S1. Distribution of MFI values for MSP-119 antibodies in Benzdorp and Stoelmanseiland. [file 12936_2020_3434_MOESM1_ESM.pptx]

## Slide 1
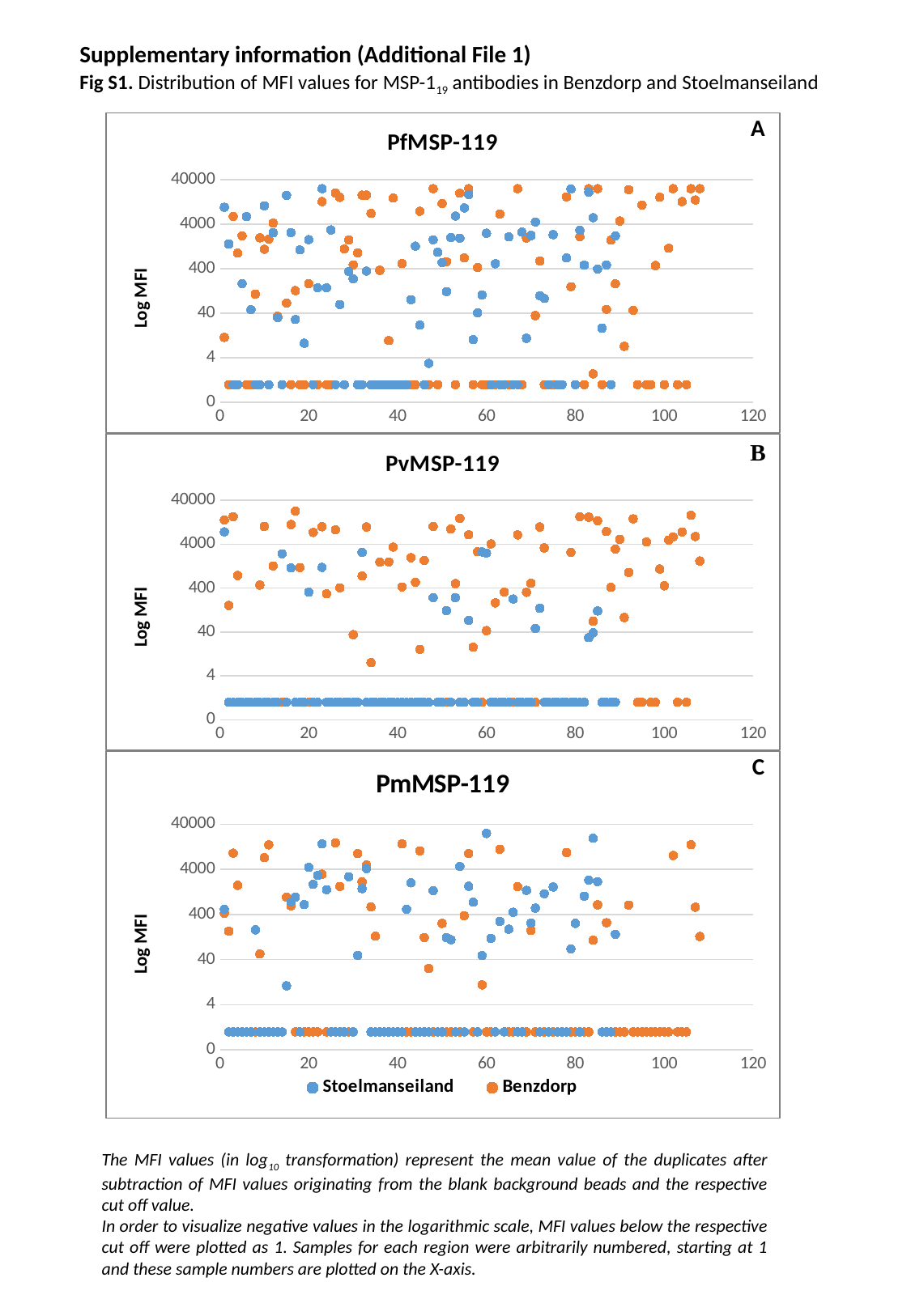

Supplementary information (Additional File 1)
Fig S1. Distribution of MFI values for MSP-119 antibodies in Benzdorp and Stoelmanseiland
### Chart: PfMSP-119
| Category | Stoelmanseiland | Benzdorp |
|---|---|---|
### Chart: PvMSP-119
| Category | Stoelmanseiland | Benzdorp |
|---|---|---|
### Chart: PmMSP-119
| Category | Stoelmanseiland | Benzdorp |
|---|---|---|The MFI values (in log10 transformation) represent the mean value of the duplicates after subtraction of MFI values originating from the blank background beads and the respective cut off value.
In order to visualize negative values in the logarithmic scale, MFI values below the respective cut off were plotted as 1. Samples for each region were arbitrarily numbered, starting at 1 and these sample numbers are plotted on the X-axis.
